# Birth-time, Etc.

**Published:** 1883-01

**Authors:** 


The Birth-time in Each Twenty-four Hours. By M. M. Walker, M. D., Germantown, Philadelphia. Reprint.

Dr. Walker takes up an idea broached by Dr. Raue some years ago, that the "incoming or flood tide" exercises some influence on parturition.

It is an old superstition (if we may so term it) that people are born as the tide flows in and die as it flows out. We cannot see that the subject is at all practical, as physicians *have* to attend the parturient female whenever sent for. And it is certain that we can't arrange our death to suit the almanac!

Homœopathic Hospital for Children, West Philadelphia.

Dr. Norton makes a good report for the fifth year of his hospital. Contributions gladly received.

